# The miR-33a-5p/CROT axis mediates ovarian cancer cell behaviors and chemoresistance *via* the regulation of the TGF-β signal pathway

**DOI:** 10.3389/fendo.2022.950345

**Published:** 2022-09-02

**Authors:** Xin Li, Xuzhu Gao, Jia Yuan, Fancheng Wang, Xiaolin Xu, Chenglong Wang, Huiqiang Liu, Wencai Guan, Jihong Zhang, Guoxiong Xu

**Affiliations:** ^1^ Research Center for Clinical Medicine, Jinshan Hospital, Fudan University, Shanghai, China; ^2^ Department of Oncology, Shanghai Medical College, Fudan University, Shanghai, China

**Keywords:** chemoresistance, microRNA, ovary, prognostic analysis, signal transduction, tumorigenesis

## Abstract

Due to the lack of symptoms and detection biomarkers at the early stage, most patients with ovarian cancer (OC) are diagnosed at an advanced stage and often face chemoresistance and relapse. Hence, defining detection biomarkers and mechanisms of chemoresistance is imperative. A previous report of a cDNA microarray analysis shows a potential association of carnitine O-octanoyltransferase (CROT) with taxane resistance but the biological function of CROT in OC remains unknown. The current study explored the function and regulatory mechanism of CROT on cellular behavior and paclitaxel (PTX)-resistance in OC. We found that CROT was downregulated in OC tissues and PTX-resistant cells. Furthermore, CROT expression was negatively correlated with the prognosis of OC patients. Overexpression of CROT inhibited the OC cell proliferation, migration, invasion, and colony formation, arrested the cell cycle at the G2/M phase, and promoted cell apoptosis. In addition, miR-33a-5p bound directly to the 3’UTR of CROT to negatively regulate the expression of CROT and promoted OC cell growth. Finally, overexpression of CROT decreased the phosphorylation of Smad2, whereas knockdown of CROT increased the nuclear translocation of Smad2 and Smad4, two transducer proteins of TGF-β signaling, indicating that CROT is a tumor suppressor which mediates OC cell behaviors through the TGF-β signaling pathway. Thus, targeting the miR-33a-5p/CROT axis may have clinical potential for the treatment of patients with OC.

## Introduction

Among gynecological malignancies, the incidence and mortality of ovarian cancer (OC) rank second in the world (1). The estimated numbers of new cases and deaths of OC are 55,342 and 37,519, respectively, in China (2), and 19,880 and 12,810, respectively, in the United States (3). Epithelial OC (EOC), accounting for more than 90% of total cases of OC, is asymptomatic at an early stage (4). Most patients (>80% of EOC) are diagnosed at an advanced stage when they visit a hospital and the survival rate of patients with EOC at an advanced stage decreases to 20% from 90% at an early stage (5, 6). The treatment of advanced OC is surgery combined with chemotherapy such as paclitaxel (PTX) and platinum (7). However, some patients develop chemoresistance and relapse (8). Hence, detecting and diagnosing OC at an early stage of the disease and preventing and reversing chemoresistance are important in order to improve patient survival and quality of life.

Carnitine O-octanoyltransferase (CROT, also known as COT), a fatty acid metabolism-related molecule, is a peroxisomal enzyme involved in fatty acid metabolism (9, 10). Most studies of CROT focus on fatty liver and atherosclerosis (11, 12). It has been reported that CROT is a potential therapeutic target for high blood pressure and stroke (12) and is associated with a decrease in the recurrence of prostate cancer analyzed by using a gene co-expression network from single-cell resolution (13). Furthermore, a cDNA microarray analysis shows that a series of genes located on chromosome 7, including *Sorcin* (*SRI)* and *CROT*, are associated with taxane resistance in OC (14). SRI is a soluble resistance-related calcium-binding protein and is involved in PTX-resistance (15). Target SRI can reverse chemoresistance *via* restoring intracellular calcium ion homeostasis (16). However, the function, effect, and regulation of CROT are not clear in OC. It has been shown that miRNAs play significant roles in the regulation of gene expression and chemoresistance (17) and CROT is a target gene of miR-33a (18, 19). Yet the expression of miR-33a in OC is unclear. In addition, cytokines such as TGF-β are important in tumorigenesis (20). TGF-β signaling has dual effects on OC progression and plasticity, suppressing OC at an early stage and promoting OC at an advanced stage (21, 22). However, the relationship between CROT and the TGF-β signaling pathway remains unknown.

The current study explored CROT as a suppressor for OC involved in PTX-resistance and cellular behaviors and its regulation by miR-33a-5p. The effect of CROT on the activation of TGF-β signaling was also examined. In addition, bioinformatics analyses were applied to define the association of the expression of CROT with a clinical prognosis and drug sensitivity.

## Materials and methods

### Cell culture

Human epithelial OC cell lines OVCAR-3, SK-OV-3, and A2780 were from American Type Culture Collection (ATCC, Manassas, VA, USA) and were cultured in Roswell Park Memorial Institute‐1640 (PRIM-1640), McCoy’5A, and Dulbecco’s modified Eagle medium (DMEM, 4.5 g/L glucose) (Corning Inc., New York, USA), respectively, supplemented with 10% fetal bovine serum (FBS, Invitrogen, Carlsbad, CA, USA). A2780-PTX cell line was from KeyGEN BioTECH (Nanjing, Jiangsu, China) and was cultured in DMEM with 10% FBS. Normal human ovarian epithelial cell line IOSE-80 and human embryonic kidney cell line 293T were from FuHeng BioLogy (Shanghai, China) and cultured in PRIM-1640 and DMEM, respectively, with 10% FBS.

### Plasmid, siRNA, miRNA, and transfection

Small-silencing RNA (siRNA) of CROT and negative control, miR-33a-5p mimics, inhibitor, and negative control were synthesized by Shanghai Gene Pharma Co., Ltd (Shanghai, China). The sequences are listed in **Supplementary Table S1**. The CROT-overexpressing plasmid was generated by inserting a coding sequence of the *CROT* gene at positions 217-2055 (GenBank Accession #: NM_021151.4) into the multi-colony site of the pcDNA4.0 vector. Wild type (WT), mutation-1 (MUT1), and mutation-2 (MUT2) of CROT 3’UTR (position 292-299) were cloned into the pmirGLO report vector for the dual-luciferase reporter assay. The X-treme GENE siRNA Transfection Reagent (Roche Applied Science, Indianapolis, USA) and Lipo8000 (Beyotime Biotechnology, Shanghai, China) were used for transfecting siRNA and plasmid, respectively.

### RNA isolation and quantitative real‐time polymerase chain reaction

Total RNA was extracted using an RNA‐Quick Purification Kit (Yishan Biotechnology Co., Ltd., Shanghai, China). The complementary DNA (cDNA) was synthesized using a First Strand Complementary DNA Synthesis kit (Roche Diagnostics, Mannheim, Germany) in a 10 μL reaction system. cDNA was then amplified using the 7300 Real-Time PCR System (Applied Biosystems; Thermo Fisher Scientific, Inc., MA, USA) with the BeyoFast™ SYBR Green qPCR Mix (2X, High ROX, Beyotime Biotechnology). Actin was used as an internal control. Primer sequences are listed in **Supplementary Table S2**.

### Protein extraction and Western blot

Total proteins were extracted using an SDS Lysis Buffer (Beyotime Biotechnology) supplemented with 1% phenylmethanesulfonylfluoride fluoride (Beyotime Biotechnology) and 1% phosphatase inhibitor (Nanjing KeyGen Biotech Co., Ltd., Nanjing, China). To separate nuclear and cytoplasmic proteins, the Minute™ SC-003 kit (Invent Biotechnologies, Inc., Beijing, China) was used according to the manufacturer’s instruction. Proteins were run on 10% SDS-PAGE and transferred into a PVDF membrane, followed by incubation with a primary antibody overnight. The following primary antibodies were used: rabbit anti-CROT (1:1000 dilution), rabbit anti-LaminB1 (1:2000 dilution), rabbit anti-Bcl-2 (1:2000 dilution), and mouse anti-GAPDH (1:5000 dilution) from Proteintech Group, Inc (Wuhan, China) and rabbit anti-Bax (1:2000 dilution), rabbit anti-Smad4 (1:2000 dilution), mouse anti-Smad2 (1:2000 dilution), rabbit anti-phospho-Smad2 (1:2000 dilution), and mouse anti-β-actin (1:5000 dilution) from Cell Signaling Technology, Inc. (Danvers, MA, USA). Secondary antibodies were horseradish peroxidase-conjugated goat anti-rabbit IgG and anti-mouse IgG (1:10,000 dilution, Proteintech). Signals were detected using BeyoECL Moon (Beyotime) and quantified using ImageJ software.

### Dual-luciferase reporter assay

After 293T cells were cultured in a 24-well-plate for 24 h at 80% confluence, 0.3 μg WT, MUT1, and MUT2 pmirGLO report plasmids were respectively transfected with 3 μl miR-33a-5p mimics, inhibitors, or negative control using Lipo8000 (Beyotime Biotechnology) for 24 h. Cells were then harvested for luciferase activity measurement using the Dual-Luciferase Reporter Gene Assay Kit (Yeasen Biotechnology, Shanghai, China). The fluorescence signals were detected by the FLUOROSKAN ASCENT FL (Thermo Scientific). After luciferase activities were measured, the ratio of firefly and Renilla luciferase intensity was calculated. Experiments were repeated at least three times.

### Immunofluorescence and immunohistochemical staining

For IF, cells were plated into glass dishes. After fixation with 4% paraformaldehyde (PFA) for 15 min, cells were permeabilized with 0.5% Triton X‐100 in phosphate-buffered saline (PBS) for 15 min. After blocking with QuickBlock™ Immunostaining Block Solution (Beyotime Biotechnology) at room temperature for 1 h, cells were incubated with the anti‐CROT antibody (1:200 Dilution, Proteintech Group, Inc), anti‐Smad2 antibody (1:100 Dilution, Cell Signaling Technology, Inc), and anti‐Smad 4 antibody (1:100 Dilution, Cell Signaling Technology, Inc) at 4°C overnight followed by the secondary antibody Alexa Fluor 594‐conjugated goat anti-rabbit IgG antibody (1:500 dilution, Cell Signaling Technology, Inc.) and Alexa Fluor 488‐conjugated goat anti-rabbit IgG antibody (1:500 dilution, Cell Signaling Technology, Inc.) incubation at room temperature for 1 h. DAPI (Beyotime) was used for the stain of nuclear. Cells were photographed under the BioTek Cytation C10 Confocal Image Reader (Agilent technologies, City, State) at ×20 or ×40 magnification.

For IHC, 10 EOC (serous type) tissues and 4 normal ovarian tissues from non-OC patients were obtained at Jinshan Hospital, Fudan University. Ethics approval was approved by the Ethics Committee of Jinshan Hospital. The 4 μm thick section of 4% paraformaldehyde-fixed, paraffin-embedded tissue specimen was deparaffinized in xylene and rehydrated in a descending alcohol series. After blocking with 10% normal goat serum for 40 min at room temperature, the section was incubated with an anti-CROT antibody (1:200 dilution, Proteintech Group, Inc) at 4°C overnight, followed by incubation with Haopoly-HRP secondary antibody according to the manufacturer’s instruction (Shanghai Jiehao Biotechnology, Inc, Shanghai, China) at room temperature for 1 h. Images were captured by OLYMPUS BX43 (OLYMPUS, Tokyo, Japan).

### Cell viability, migration, invasion, colony formation, EdU, and cell cycle assays

For the cell viability assay, CROT overexpressing plasmids or siRNA-transfected OVCAR-3, SK-OV-3, and A2780 cells were seeded in 96-well plates at the density of 4×10^3^, 5×10^3^, and 3×10^3^ per well, respectively, for 24, 48, 72, and 96 h. Absorbance values for cells were measured using the CCK8 kit (Beyotime) at 450nm.

For the migration assay, cells were directly plated into the upper chamber of a Transwell (Corning Inc.). For the invasion assay, the Transwell was firstly coated with 5% Matrigel (BD Biosciences, New Jersey, USA) and incubated at 37 ^0^C for at least 8 h before cell plating. After OVCAR-3, SK-OV-3, and A2780 cells were added to the upper chamber at the density of 3×10^4^, 5×10^4^, and 8×10^4^ per well, respectively, the lower chamber was filled with a medium containing 20% FBS, followed by incubation for 48 h. Cells were then fixed and stained with Crystal violet solution (Sigma) for 30 min. The cells on the upper side of the chamber were removed, whereas the cells on the bottom side of the chamber were photographed at ×20 magnification using a light microscope (OLYMPUS, Tokyo, Japan). Finally, the number of cells was analyzed by Image J software.

For colony formation, cells were plated into 6-well plates at a density of 1×10^3^/well. After 2 weeks, cells were fixed with 4% PFA for 30 min and stained with Crystal violet solution (Sigma) for 30 min. The number of colonies was analyzed using Image J software.

For the EdU proliferation assay, after seeding cells in a 24-well plate for 24 h, cells were labeled by using the BeyoClick™ EdU-555 kit (Beyotime) according to the manufacturer’s instruction. Cells were then photographed under the BioTek Cytation C10 Confocal Image Reader (Agilent Technologies) at ×20 magnification. The ratio of red/blue was analyzed using Image J software.

For cell cycle analysis, cells were seeded in a 6-well plate for 24 h. After trypsinization, cells were washed using cold PBS twice at 1,000 rpm for 5 min each and fixed with 70% ethanol at -20°C for 4 h, followed by washing with PBS twice. The cell pellet was then resuspended in 500 µl propidium iodide (PI) solution (PI/RNase Staining Buffer, BD, NJ, USA) and incubated for 15 min in dark. Fifteen thousand cells were acquired by flow cytometry (Beckman Coulter, Inc., Brea, CA, USA). Data were analyzed using ModFit™ software.

### Wound-healing assay

OVCAR-3 and SK-OV-3 cells were plated into 12-well plates and grew to 95% confluence. A sterile 200-μL pipette tip was used to scratch cells to make a wound. After cells were cultured in a serum-free medium, images were captured at 0 and 72 hours, respectively, using the phase-contrast microscope (OLYMPUS).

### Apoptosis assay

Cells were cultured in 6-well plates for 24 h. After harvesting, cells were washed with PBS and resuspended in 500 µl 1× binding buffer, 3 µl Annexin V (BD) and 5 µl PI were added to the cell suspension. After incubation for 15 min, the apoptotic cells were measured by flow cytometry (Beckman Coulter, Inc.).

### GEO data resource and clinical correlation analyses

Gene Expression Omnibus (GEO) expression profiling datasets (GSE26712, GSE18521, GSE40595, GSE38666, GSE14407, and GSE52460) containing normal ovarian samples and OC samples were downloaded from https://www.ncbi.nlm.nih.gov/geo/. Detailed information on the GEO series was summarized in **Supplementary Table S3**. Overall survival (OS) of patients with the high/low expression of CROT was analyzed based on the data from GSE3149 and GSE15622 using “survival”, “survminer”, “limma”, and “ggpubr” R-packages. The online UALCAN database (http://ualcan.path.uab.edu) was used to predict the correlation between CROT expression and clinical features.

### Co-expression gene analysis and gene set enrichment analysis of CROT

The co-expression genes of CROT in OC were acquired from GSE26712 and were presented as heatmap and volcano map using “limma”, and “heatmap” R packages. The Database for Annotation, Visualization and Integrated Discovery (DAVID 6.8) (23) was used for the Gene Ontology (GO) enrichment analysis and Kyoto Encyclopedia of Genes and Genomes (KEGG) pathway enrichment analysis based on the above co-expression genes of CROT. The enrichment results were then visualized using a “ggplot2” package with a p-value <0.05. GO enrichment analysis included biological processes (BP), cellular components (CC), and molecular function (MF) analysis. Metascape (http://metascape.org) was used for gene annotation and enrichment analysis. The ‘Express Analysis’ module of Metascape was used to further verify the enrichment of CROT and closely related neighbor genes. The GSEA of CROT was performed based on the data from TCGA-OV and GTEx. A p-value <0.05 and a false discovery rate (FDR) <0.25 were considered as significance.

### Drug sensitivity analysis in cancer

NCI-60 compound activity data and RNA-seq expression profiles were acquired and downloaded from the CellMiner™ database (https://discover.nci.nih.gov/cellminer/home.do). The “impute”, “limma”, “ggplot2”, and “ggpubr” R packages were performed to analyze the correlation between CROT expression and the half-maximal inhibitory concentration (IC_50_) of chemotherapy drugs.

### Statistical analysis

All data were analyzed by GraphPad Prism 8.0 (GraphPad Software Inc.) and presented as the mean ± standard error of the mean (SEM). The Student’s *t*‐test was used for two‐group comparison. One-Way ANOVA was used for multiple comparisons. The Wilcoxon rank-sum test and Pearson Correlation Coefficient were applied to analyze the difference and correlation between the two groups, respectively. The Kaplan-Meier survival curve analysis was performed using the Log-rank test. The Cox hazard regression model was applied to calculate the hazard ratio (HR). Statistical significance was considered when P < 0.05.

## Results

### CROT expression is low in ovarian cancer and paclitaxel-resistant cells

**Figure 1:**
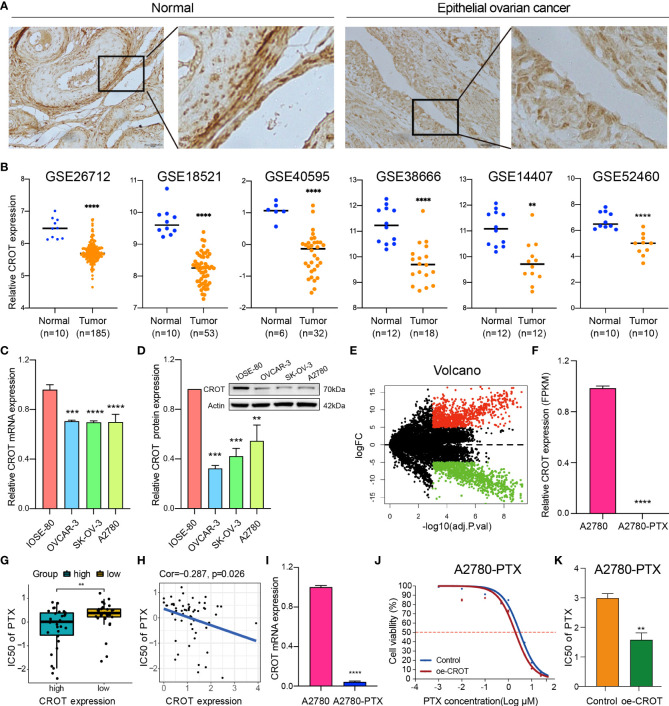
Abnormal expression of CROT in ovarian cancer and paclitaxel-resistant cells. **(A)** The expression of CROT was detected by IHC. Representative images in normal epithelial ovarian tissues (n=4) and epithelial ovarian cancer tissues (n=10) are shown. Magnification, ×400; Scale bar, 50 μm. **(B)** Expression levels of CROT between normal and tumorous epithelial ovarian tissues from GEO datasets (GSE26712 and GSE18521 were analyzed by using the Wilcoxon rank-sum test, whereas GSE40595, GSE138666, GSE14407, and GSE52460 were analyzed by using Student’s *t*-text). Counts of each sample were transformed by Log2. **(C, D)** Detection of CROT expression in EOC cells (OVCAR-3, SK-OV-3, and A2780) and non-tumorous immortalized ovarian surface epithelial cells (IOSE-80) at mRNA and protein levels by qRT-PCR and Western blot. One-way ANOVA was applied **(E)** Volcano map showed 1747 differentially expressed genes (834 up and 913 down) using RNA-seq data of PTX-resistant OC cells A2780-PTX and its counterpart sensitive OC cells A2780 (P<0.001, logFC>5). **(F)** Measurement of CROT expression in A2780 and A2780-PTX cells by the fragments per kilobase of exon per million fragments mapped (FPKM). **(G)** Analysis of the IC_50_ of PTX between high and low expression levels of CROT using the data from the CellMiner database. **(H)** Correlation analysis (Pearson Correlation Coefficient) between the IC_50_ of PTX and expression levels of CROT. **(I)** Detection of CROT mRNA expression in A2780-PTX and A2780 cells by qRT-PCR. **(J)** Measurement of A2780-PTX cell viability after overexpression of CROT and treatment of PTX by the CCK8 assay. **(K)** Calculation of the IC_50_ of PTX in A2780-PTX cells. Assays were repeated at least three times. Data were presented as mean ± SEM. CROT, Carnitine O-octanoyltransferase; IC_50_, the half-maximal inhibitory concentration; PTX, paclitaxel; *p < 0.05; **p < 0.01; ***p < 0.001; ****p < 0.0001.

The expression of CROT was examined in normal ovarian and EOC tissues/cells. IHC showed that CROT expression was lower in EOC tissues (n=10 patients) than in normal ovarian tissues (n=4) **(** **Figure 1A** **).** The low expression of CROT in OC was confirmed after bioinformatics analyses of the data from RNA-seq datasets of GSE26712, GSE18521, GSE40595, GSE38666, GSE14407, and GSE52460 (**Figure 1B** **)**. Furthermore, the decrease in CROT expression was also observed in EOC cells (OVCAR-3, SK-OV-3, and A2780) compared to non-tumorous immortalized ovarian surface epithelial cells (IOSE-80) at mRNA and protein levels by qRT-PCR and Western blot (**Figures 1C, D**). The IF assay showed that the signal of CROT staining was weaker in SK-OV-3 and A2780 cells than in IOSE-80 cells and CROT was distributed both in the cytoplasm and nucleus (**Supplementary Figure S1**). Next, we examined whether CROT was differentially expressed between PTX-resistant OC cells A2780-PTX and its counterpart sensitive OC cells A2780 (24). RNA-seq analysis showed 1747 differentially expressed genes (834 up and 913 down) as presented in the volcano map (P<0.001, logFC>5) (**Figure 1E**), in which CROT was detectable in A2780 cells and almost undetectable in A2780-PTX cells measured by using the fragments per kilobase of exon per million fragments mapped (FPKM) (**Figure 1F**). In addition, the impact of CROT on the IC_50_ of PTX was analyzed using the data from the CellMiner database. The statistical analysis showed an increase in the IC_50_ of PTX in CROT-low cells compared to CROT-high cells (**Figure 1G**). Correlation analysis (Pearson) showed a negative association between the IC_50_ of PTX and expression levels of CROT (**Figure 1H**). Using qRT-PCR, we detected the downregulation of CROT expression in A2780-PTX cells compared to A2780 cells (**Figure 1I**), which was consistent with the results of our RNA-seq data analyzed, whereas the overexpression of CROT in A2780-PTX cells significantly declined the IC_50_ of PTX (**Figures 1J, K**), indicating that CROP may increase the PTX sensitivity.

### Low expression of CROT is correlated with poor prognosis

**Figure 2:**
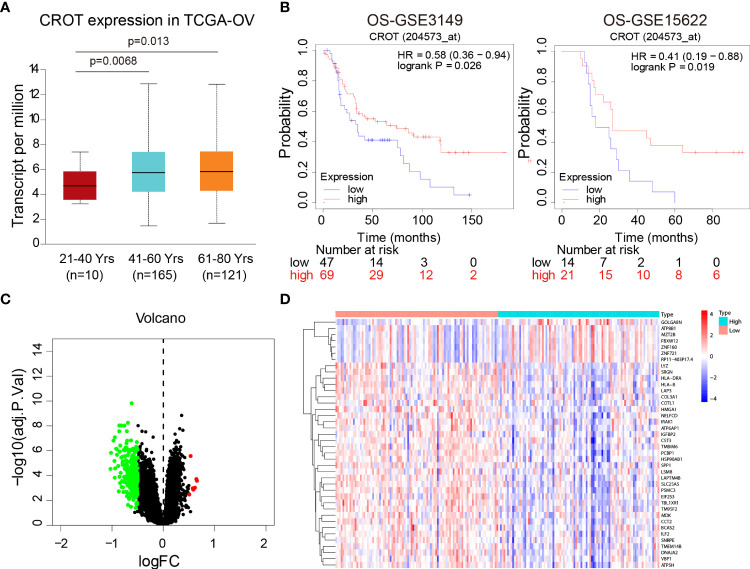
Association of CROT expression with prognosis. **(A)** Association of CROT expression with ages of OC patients based on the data from the TCGA-OV dataset using the UALCAN database. **(B)** The correlation of CROT expression with the overall survival (OS) of patients with OC was analyzed by the Kaplan-Meier plotter. The data were from GSE3149 and GSE15622 datasets. **(C, D)** Co-expressed genes with CROT presented in volcano map and heatmap. The data were from the GSE26712 dataset (logFC>0.5, p<0.05). TCGA-OV, The Cancer Genome Atlas ovarian cancer.

Since CROT expression was low in OC and was related to PTX resistance, we next analyzed the association of CROT expression with the clinical features of OC patients. The expression of CROT was lower in younger patients (21-40 years) than in middle-aged (41-60 years) and elderly patients (61-80 years) (**Figure 2A**). Subsequently, the Kaplan-Meier plotter showed that low expression of CROT was correlated with a poor prognosis of patients with OC based on data from GSE3149 and GSE15622 datasets (**Figure 2B**). To figure out the biological functions of CROT in OC, GO and KEGG enrichment analyses were performed based on co-expressed genes with CROT from the GSE26712 dataset. Co-expressed genes were shown in the volcano map and heatmap (**Figures 2C, D**). Using the same dataset, we performed GO term and KEGG enrichment analyses to get more information about the functions of CROT in OC. GO term annotation showed that co-expressed genes of CROT were primarily involved in the apoptotic process, response to drug, focal adhesion, and protein binding (**Supplementary Figures S2A–C**). KEGG pathway analysis showed that CROT expression was mainly enriched in the cell adhesion, cell cycle, and DNA replication (**Supplementary Figure S2D**). Moreover, enrichment analysis using Metascape showed enriched pathways of CROT including cellular process, regulation of the biological process, immune system process, signaling, and growth (**Supplementary Figure S3**).

### CROT inhibits cell proliferation and arrests the cell cycle

**Figure 3:**
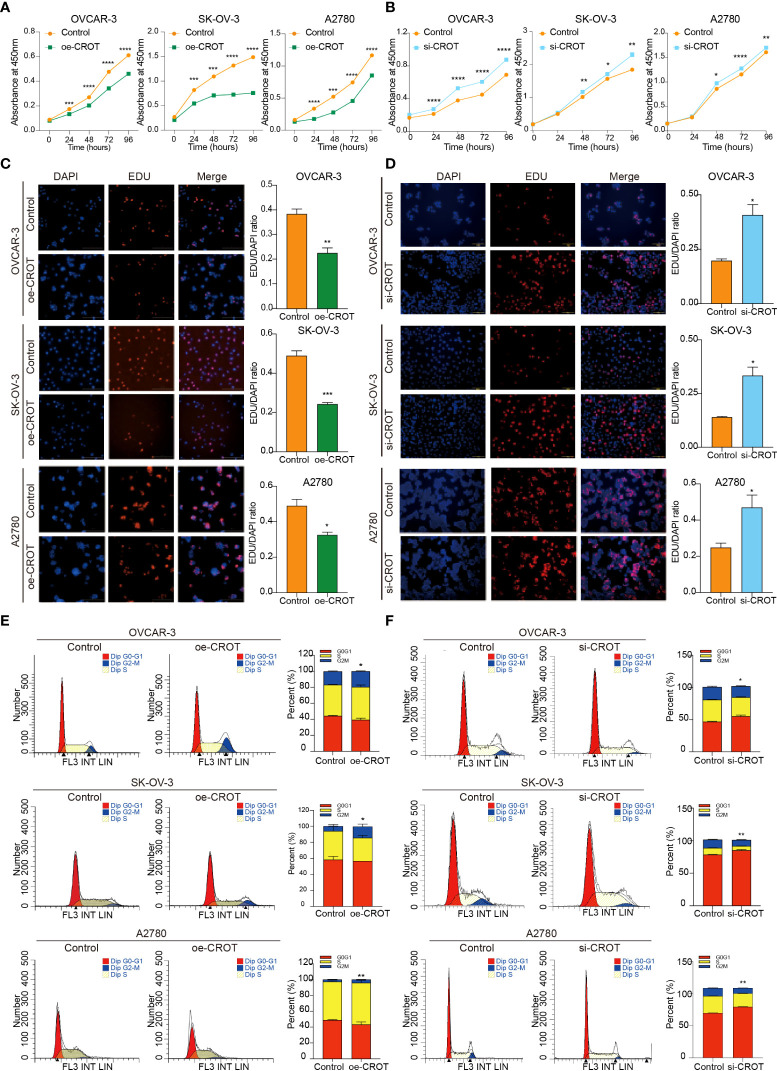
Effect of CROT on cell proliferation and cell cycle in OVCAR-3, SK-OV-3, and A2780. **(A**, **B)** Detection of cell viability by the CCK-8 assay after CROT-overexpressing plasmid (oe-CROT) and CROT-siRNA (si-CROT) transfection for 0, 24, 48, 72, and 96 h. **(C**, **D)** The EdU assay and statistical analysis after oe-CROT and si-CROT transfection for 48 h. **(E**, **F)** The cell cycle detection by flow cytometry and statistical analysis after oe-CROT and si-CROT transfection for 48 h. Assays were repeated at least three times. Data were presented as mean ± SEM. *P <0.05; **P < 0.01; ***P < 0.001.

Since CROT was downregulated in OC and bioinformatics analysis showed involvement of CROT in the cell cycle, next we examined the effect of CROT on cellular behaviors. To approach a gain-of-function of CROT in OC cells, we constructed a CROT-overexpressing plasmid. qRT-PCR and Western blot analyses confirmed the overexpression of CROT in OVACR-3, SK-OV-3, and A2780 cells at mRNA and protein levels after the transfection of CROT-overexpressing plasmid (**Supplementary Figures S4A**–**C**). Contrarily, we performed a loss-of-function approach using CROT-siRNA. We found that 2 CROT-siRNAs sufficiently knocked down CROT expression in OC cells at mRNA and protein levels detected by qRT-PCR and Western blot (**Supplementary Figures S4D**–**F**). Overexpression of CROT decreased, whereas knockdown of CROT increased, OC cell viability measured by the CCK-8 assay (**Figures 3A, B**). In addition, the EdU assay verified that overexpression of CROT decreased, whereas knockdown of CROT increased, the DNA replication in OVACR-3, SK-OV-3, and A2780 cells (**Figures 3C, D**). Finally, the cell cycle analysis showed that the change of CROT expression mainly affected the G2/M phase in OC cells (**Figures 3E, F**), endowing the function of CROT on cell proliferation. These data indicate that the loss of CROT may lead to OC cell growth.

### CROT inhibits migration, invasion, and colony formation

**Figure 4:**
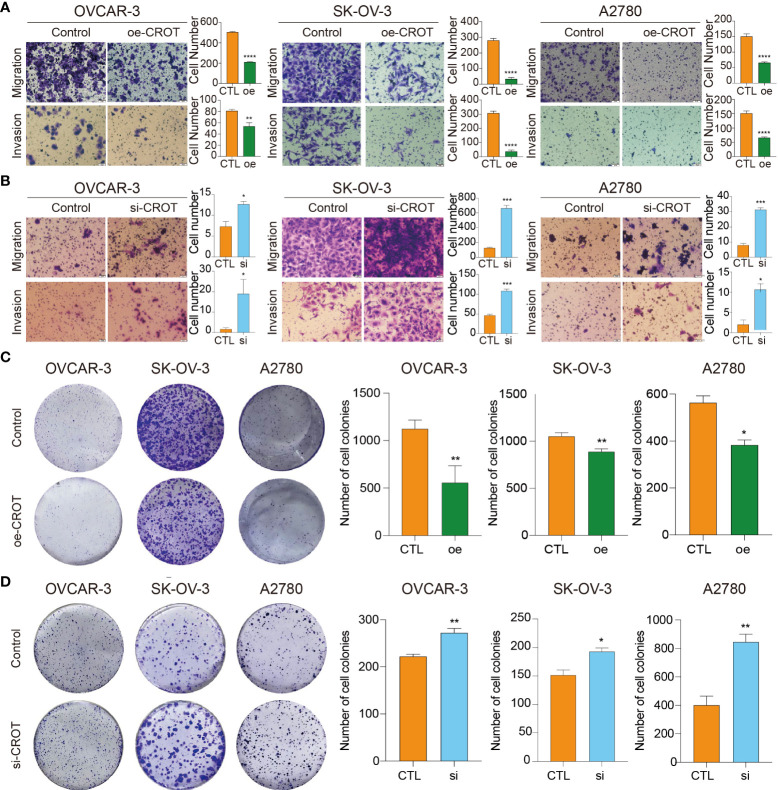
Detection of migration, invasion, and colony formation in OVCAR3, SKOV3, and A2780 cells. **(A)** Detection of cell migration and invasion after CROT-overexpressing plasmid (oe-CROT) transfection for 48 h. **(B)** Detection of cell migration and invasion after CROT-siRNA (si-CROT) transfection for 48 h. **(C)** Detection of colony formation after CROT-overexpressing plasmid (oe-CROT) transfection for 2 weeks. **(D)** Detection of colony formation after CROT-siRNA (si-CROT) transfection for 2 weeks. Histograms show the statistical analyses. Assays were repeated at least three times. Data presented as mean ± SEM. *P <0.05; **P < 0.01; ***P < 0.001; ****P < 0.001; oe, overexpression of CROT; si, CROT-siRNA; CTL, negative control.

The overexpression of CROT inhibited OC cell migration and invasion (**Figure 4A**), whereas the knockdown of CROT stimulated OC cell migration and invasion (**Figure 4B**). In addition, the wound healing assay showed slow migration after CROT-overexpressing plasmid transfection (**Supplementary Figure S5A**) and fast migration after CROT-siRNA transfection (**Supplementary Figure S5B**) compared to their negative controls in OVACR-3 and SK-OV-3 cells. The overexpression of CROT significantly suppressed colony formation (**Figure 4C**), whereas the knockdown of CROT significantly accelerated colony formation (**Figure 4D**) in OVCAR3, SKOV3, and A2780.

### CROT promotes cell apoptosis

**Figure 5:**
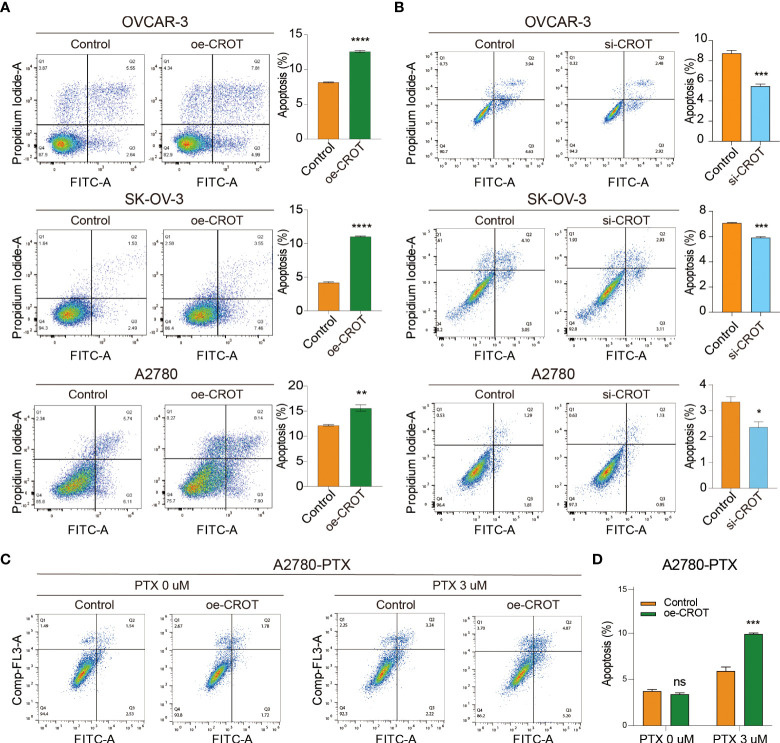
Measurement of apoptotic cells by flow cytometry. **(A)** Detection of apoptotic cells after CROT-overexpressing plasmid (oe-CROT) transfection in OVCAR-3, SK-OV-3, and A2780 for 48 h. **(B)** Detection of apoptotic cells after CROT-siRNA (si-CROT) transfection in OVCAR-3, SK-OV-3, and A2780 for 48 h. **(C)** Chemoresistant A2780-PTX cells were transfected with oe-CROT for 48 h, followed by 3 μM PTX treatment for 48 h. **(D)** The histogram shows the statistical analyses of **(C)** Assays were repeated at least three times. Data presented as mean ± SEM. *P <0.05; **P < 0.01; ***P < 0.001; ****P < 0.001; PTX, paclitaxel.

Next, we examined the effect of CROT on cell apoptosis. Overexpression of CROT promoted, whereas knockdown of CROR inhibited, apoptosis significantly in OVCAR-3, SK-OV-3, and A2780 cells detected by the flow cytometry (**Figures 5A, B**). Western blot showed an increase in Bax protein and a decrease in Bcl-2 protein in SK-OV-3 and A2780 cells after CROT overexpression (**Supplementary Figures S6A, B**). Furthermore, apoptosis was increased after the treatment of PTX (3 μm) in the presence of a CROT-overexpressing plasmid in A2780-PTX cells (**Figures 5C, D**), indicating that CROT may enhance PTX sensitivity in terms of promoting apoptosis.

### CROT is regulated by miR-33a-5p

**Figure 6:**
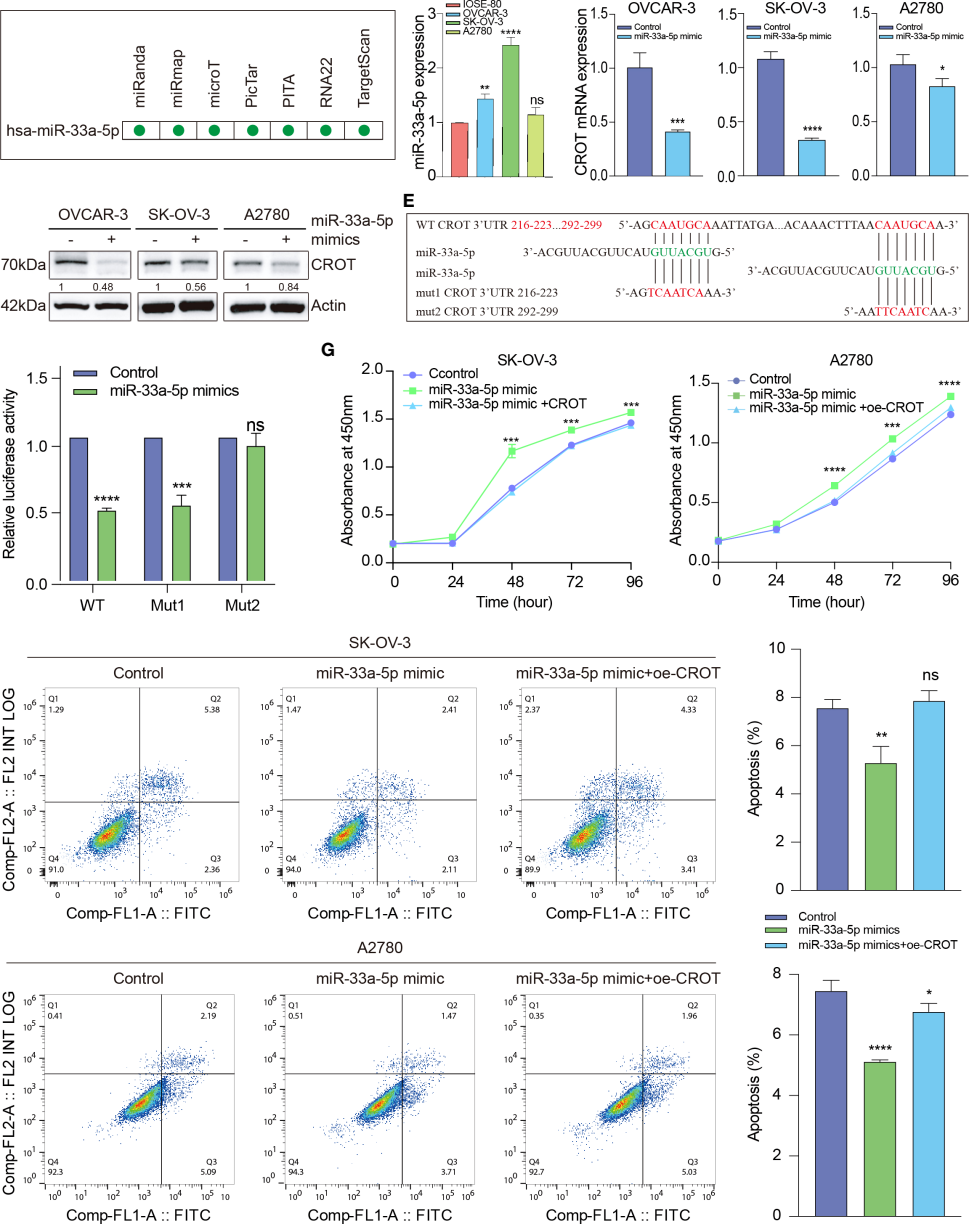
The function of miR-33a-5p in ovarian cancer cells. **(A)** Prediction of hsa-miR-33a-5p by the Encyclopedia of RNA Interactomes (ENCORI) online platform, including miRanda, miRmap, microT, PicTar, PITA, RNA22, and TargetScan programs. **(B)** Detection of the expression of miR-33a-5p in OC cells (OVCAR-3, SK-OV-3, and A2780) and non-tumorous ovarian epithelial cells (IOSE-80) by qRT-PCR. **(C, D)** Detection of CROT mRNA and protein in OVCAR-3, SK-OV-3, and A2780 cells after the treatment of cells with miR-33a-5p mimics by qRT-PCR and Western blot. **(E)** Match of two potential binding sites of miR-33a-5p to the 3’UTR of CROT using the TargetScan database. The sequences of wild-type and 2 mutants of the 3’UTR of CROT were shown. **(F)** Detection of luciferase activity. 293T cells were transfected with luciferase report plasmids (WT, Mut1, or Mut2), followed by the treatment of miR-33a-5p mimics. The luciferase activity was detected by the dual-luciferase assay. **(G)** Measurement of cell viability. SK-OV-3 and A2780 cells were treated with miR-33a-5p mimics in the presence or absence of CROT-overexpressing plasmids. The cell viability was measured by the CCK-8 assay. **(H, I)** Detection of apoptotic cells. SK-OV-3 and A2780 cells were treated with miR-33a-5p mimics in the presence or absence of CROT-overexpressing plasmids. Apoptotic cells were detected by flow cytometry. Histograms show the statistical analyses of the data. Assays were repeated at least three times. Data were presented as mean ± SEM. *P <0.05; **P < 0.01; ***P < 0.001; ****P < 0.0001; ns, not significant; WT, wild type; Mut1, mutant 1; Mut2, mutant 2; 3’UTR, 3’ untranslated region; oe-CROT, overexpression of CROT.

To further investigate the mechanism underlying the downregulation of CROT in OC cells and since miRNAs can be upstream regulators of CROT, an ENCORI online platform was used to search candidates (25). We found that hsa-miR-33a-5p was predicted to have potentially binding ability to the CROT mRNA by miRanda, miRmap, microT, PicTar, PITA, RNA22, and TargetScan programs (**Figure 6A**). Next, we found that the expression of miR-33a-5p was higher in OC cells (OVCAR-3, SK-OV-3, and A2780) than in non-tumorous ovarian epithelial cells (IOSE-80) detected by qRT-PCR (**Figure 6B**). Treating OC cells with miR-33a-5p mimics resulted in a downregulation of CROT expression at the mRNA and protein levels in OVCAR-3, SK-OV-3, and A2780 cells detected by qRT-PCR and Western blot (**Figures 6C, D**). Contrarily, the treatment of miR-33a-5p inhibitors led to an increase in CROT expression in SK-OV-3 cells (**Supplementary Figures S7A, B**). Subsequently, we matched two potential binding sites of miR-33a-5p to the 3’UTR of CROT using the TargetScan database and constructed luciferase report plasmids. The sequences of wild-type and 2 mutants of the 3’UTR of CROT were shown (**Figure 6E**). Dual-luciferase assays indicated that miR-33a-5p mimics decreased luciferase activity in 293T cells after wild-type and mut1-plasmid transfection but not mut2-plasmid transfection (**Figure 6F**), suggesting the direct binding of miR-33a-5p mimics with the 3’UTR site-2 of CROT. Furthermore, the treatment of miR-33a-5p mimics increased the SK-OV-3 and A2780 cell survival, whereas overexpression of CROT diminished miR-33a-5p mimics-increased cell survival (**Figure 6G**). Moreover, miR-33a-5p was found to inhibit SK-OV-3 and A2780 cell apoptosis, which was abolished after the overexpression of CROT (**Figures 6H, I**).

### CROT regulates TGF-β signal transducer proteins

**Figure 7:**
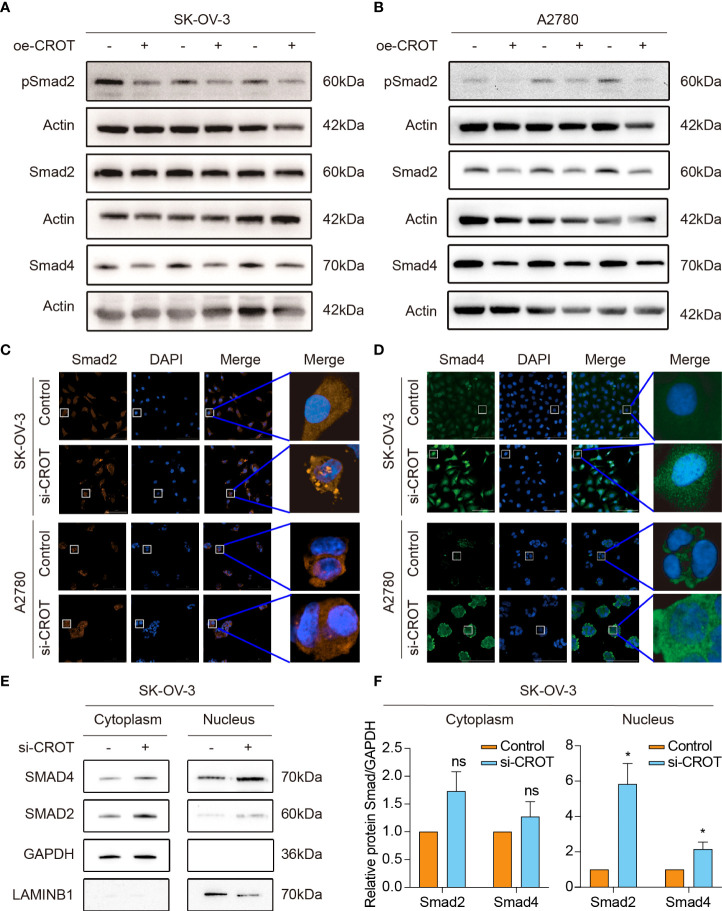
Effect of CROT on the TGF-β signaling pathway. **(A, B)** Detection of Smad proteins by Western blot in SK-OV-3 and A2780 cells transfected with CROT-overexpressing plasmid (oe-CROT) for 48 h. **(C, D)** Detection of Smad2 and Smad4 signals by immunofluorescence staining after CROT-siRNA (si-CROT) transfection in SK-OV-3 and A2780 for 48 h. Original magnification, ×400. **(E)** Detection of Smad 2 and Smad4 proteins in the cytoplasm and nucleus of SKOV3 cells transfected CROT-siRNA (si-CROT) for 48 h by Western blot. **(F)** Histograms show the statistical analyses of E. Data were presented as mean ± SEM (n=3). *P <0.05; ns, not significant.

Our previous study showed that TGF-β signaling has a significant effect on OC cell behaviors (26, 27), here we performed a GSEA analysis of the data of OC samples from the TCGA-OV database and normal ovarian samples from the GTEx database to see whether high or low expression of CROT is associated with the TGF-β signaling pathway. Interestingly, the TGF-β signaling pathway was found to be negatively correlated with CROT expression (**Supplementary Figure S8A**). However, the administration of TGF-β1 (1 or 10 ng/mL) did not affect CROT expression at mRNA and protein levels (**Supplementary Figures S8B, C**). Subsequently, we examined the regulatory effect of CROT on the TGF-β signal transducer proteins. Overexpression of CROT decreased the phosphorylation of Smad2 and the level of Smad4 expression in SK-OV-3 and A2780 cells detected by Western blot (**Figures 7A, B**). Furthermore, the knockdown of CROT resulted in the nuclear translocation of Smad2 and Smad4 detected by the IF assay (**Figures 7C, D**), which was confirmed by Western blot that the levels of Smad 2 and Smad4 were increased in the nucleus after CROT-siRNA transfection (**Figures 7E, F**). These data indicate that CROT may negatively regulate the TGF-β signaling pathway.

## Discussion

The current study examined the expression and function of CROT in OC and explored the potential mechanisms underlying CROT-mediated cell behaviors and its regulation in OC cells. Our data proved that OC had low expression of CROT and the expression levels were associated with the prognosis of patients. Furthermore, we found that CROT was regulated by miR-33a-5p and affected TGF-β signal transducers.

It has been shown that cancer cells with molecular and cellular programming can facilitate survival and colonization (28). CROT is an enzyme involved in lipid metabolism and fatty acid oxidation (9, 29). In the scientific literature, most studies focus on the function of CROT in vascular disease (10, 12). However, the effect of CROT on cancer progression is not clear. Here, we demonstrated for the first time that the expression of CROT was lower in OC. Overexpression of CROT changed cell behaviors, leading to a decrease in cell proliferation, migration, invasion, and colony formation. These data indicate that CROT acts as a tumor suppressor. Furthermore, overexpression of CROT promoted, whereas knockdown of CROT inhibited, OC cell apoptosis detected by flow cytometry. We also observed that overexpression of CROT increased Bax and decreased Bcl-2. Bax is a pro-apoptotic protein (30) and Bcl-2 is an outer mitochondrial membrane protein that is known to block apoptosis (31). The alteration of CROT affects OC cell apoptosis, driving at least in part *via* the regulation of Bax/Bcl-2. Targeting CROT may be a good strategy in OC treatment.

Interestingly, our study found that CROT expression was related to PTX-resistance. CROT was almost undetectable in A2780-PTX cells but highly detectable in A2780 cells, which was confirmed by Correlation analysis (Pearson) and RNA-seq data analysis. We also observed a negative association between the expression levels of CROT and the IC_50_ of PTX, indicating that CROT may increase the PTX sensitivity. A previous report showed that *CROT*, together with a cluster of genes (*MDR1*, *SRI*, *MGC4175*, *CLDN12*, and *CDK6*) located on chromosome 7, is identified as a gene for acquired resistance of taxanes in six OC cell lines by cDNA microarray analysis (14). The *MDR1* gene is a well-known multidrug resistance gene (32). Our group recently reported that SRI is a key driver of PTX-resistance medicated *via* Smad4/ZEB1/miR-142-5p in human OC (15). Here, we further demonstrated that *CROT* is another resistant-related gene. Overexpression of CROT inhibited OC cell growth which might confer CROT as a sensitizer of PTX.

The prognosis of patients with OC does not get much improved although treatments have been developed and improved over decades (33). As mentioned above, patients with OC are often diagnosed at an advanced stage because of lacking typical and visible symptoms. Although the previous study reported that there is no difference in the OS between younger (<65 years) and elderly (≥65 years) patients with OC and claimed that age is not an independent prognostic factor (34), the current study by analyzing the data from TCGA-OV showed that the expression of CROT was lower in younger patients (<40 years) than in middle-aged (41-60 years) and elderly (>60 years) patients and OC patients with low expression CROT had worse outcomes compared to patients with higher CROT. These data indicate that the CROT has the potential to be a new prognostic factor. In other words, the high level of CROT expression is in favor of a good prognosis in patients with OC. The size of clinical samples should be increased and more patients should be recruited for future studies. It has been shown that an increase in CROT activity induces a decrease in very long chain fatty acids levels (9). Patients who have a low expression of CROT may suffer from the disorder of fatty acid metabolism because fatty acids support tumorigenesis and metastasis (35) and therefore, leading to poor prognoses. Furthermore, cellular fatty acid metabolic changes are related to drug resistance through enhanced lipid synthesis, storage, and catabolism (36).

Our GO enrichment analysis showed that CROT was closely associated with collagen and focal adhesion which may account for the inhibitory effect of CROT on migration and invasion of OC. The detailed mechanism underlying the effect of CROT on cell behaviors is unclear. The current study demonstrated that miR-33a-5p was upregulated in OC and was an upstream regulator of CROT. The level of miR-33a-5p was negatively associated with the expression of CROT in OC. Administration of miR-33a-5p mimics promoted OC cell survival and reduced OC cell apoptosis, which was abolished in the presence of the overexpression of CROT. Many studies report that miR-33a is involved in fatty acid/lipid metabolism and metabolic diseases (37–39). Cancer is also considered a metabolic disease (40) and the metabolic disorder has been associated with poor outcomes in EOC (41). Therefore, we presumed that the miR-33a-5p/CROT axis plays an important role in mediating OC cell behaviors. Furthermore, the positive regulation of TGF-β on fatty acid oxidation has been observed in breast and colorectal cancer cells (42, 43), implying that TGF-β may also be involved in CROT-mediated OC cell behaviors. Indeed, GSEA enrichment analysis showed the negative relationship between CROT and TGF-β signaling pathway. TGF-β is a cytokine and many studies have shown that TGF-β transduction is dysregulated in cancer (20, 44). Our experiments proved the influence of CROT on TGF-β signaling proteins Smad2 and Smad4 rather than the regulation of CROT by the TGF-β signaling pathway. We thought that CROT mediated cell behaviors at least in part *via* the regulation of Smad2/4, interfering with the TGF-β signaling pathway to affect cell proliferation. Targeting the TGF-β signaling pathway in cancer treatment has been suggested (45) and hence, along with the line of miR-33a-5p/CROT/Smad proteins, a better therapeutical strategy should be designed for OC patients.

**Figure 8:**
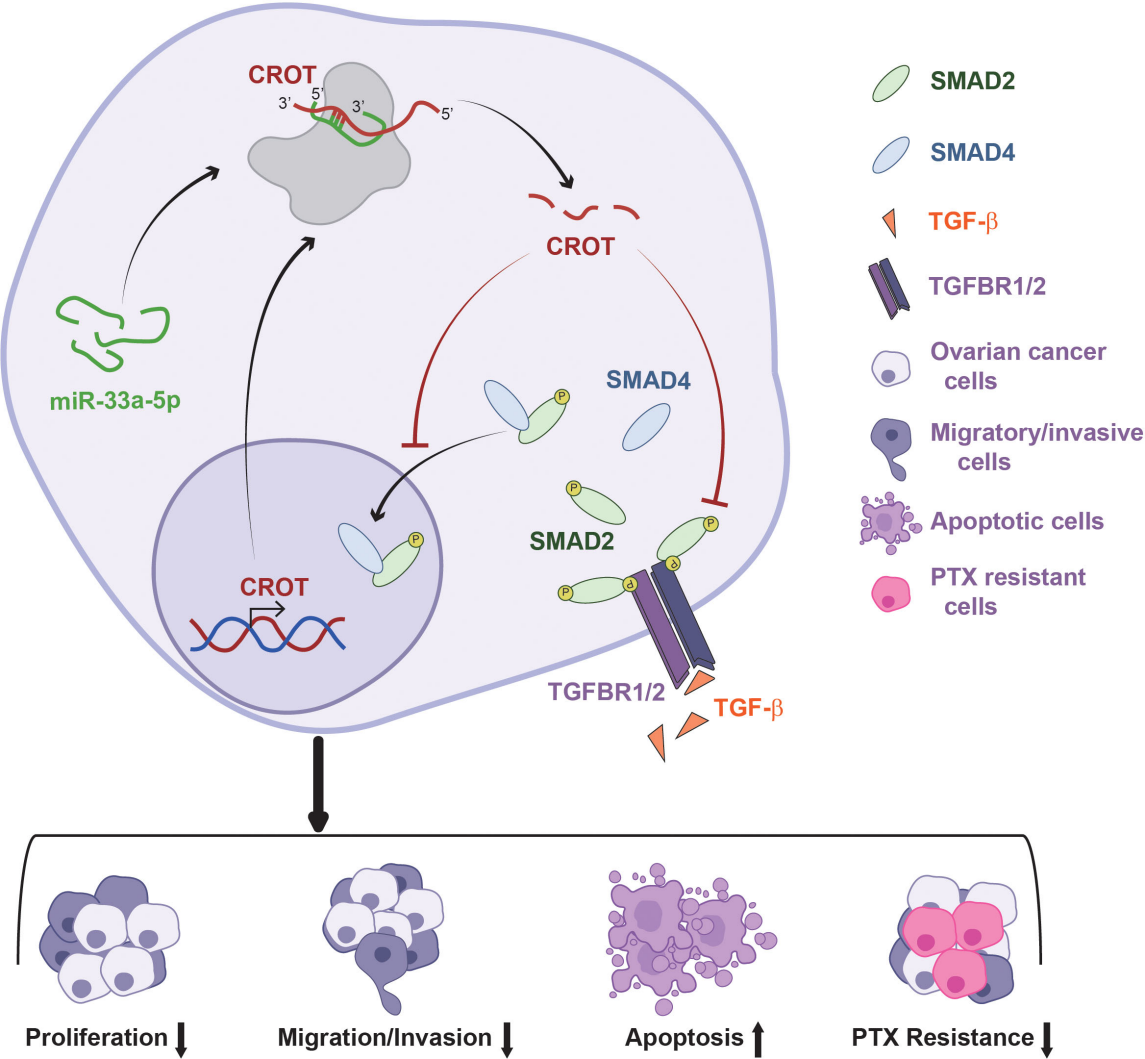
Schematic model illustrates the regulatory mechanism of the miR-33a-5p/CROT axis mediating ovarian cancer cell behaviors and chemoresistance *via* the regulation of the TGF-β signal pathway.

In conclusion, CROT expression is downregulated in OC tissues and PTX-resistant OC cells and functions as a tumor suppressor which affects OC cell behaviors and the prognosis of OC patients. The expression of CROT is regulated by miR-33a-5p which is upregulated in OC cells. In addition, CROT negatively regulates TGF-β signal transducer proteins Smad2 and Smad4 (**Figure 8**). Thus, the miR-33a-5p/CROT axis mediates cell behaviors partially *via* the regulation of TGF-β signal transducer proteins. Targeting miR-33a-5p/CROT/Smads might be helpful for the diagnosis, treatment, and prognosis of patients with OC.

## Data availability statement

The datasets generated for this study can be found in online repositories. The names of the repository/repositories and accession number(s) can be found in the article/**Supplementary Material**.

## Ethics statement

The studies involving human participants were reviewed and approved by Ethics approval was approved by the Ethics Committee of Jinshan Hospital. Written informed consent for participation was not required for this study in accordance with the national legislation and the institutional.

## Author contributions

XL and XG performed the experiments, analyzed data, and wrote the draft of the manuscript. JY, FW, XX, CW, and HL did partial experiments and validated and analyzed data. WG and JZ performed cell culture and provided technical support. GX did supervision, conceptualization, and project administration, and wrote and edited the final manuscript. All authors contributed to the article and approved the submitted version.

## Funding

This work was supported by the National Natural Science Foundation of China (No. 81872121) and the Science and Technology Commission of Shanghai Municipality (No. 17ZR1404100) to GX.

## Conflict of interest

The authors declare that the research was conducted in the absence of any commercial or financial relationships that could be construed as a potential conflict of interest.

## Publisher’s note

All claims expressed in this article are solely those of the authors and do not necessarily represent those of their affiliated organizations, or those of the publisher, the editors and the reviewers. Any product that may be evaluated in this article, or claim that may be made by its manufacturer, is not guaranteed or endorsed by the publisher.
