# Supplementary material for: The miR-33a-5p/CROT axis mediates ovarian cancer cell behaviors and chemoresistance via the regulation of the TGF-β signal pathway
Source: Front Endocrinol (Lausanne). 2022 Sep 2;13:950345. doi: 10.3389/fendo.2022.950345 (PMC9478117; doi:10.3389/fendo.2022.950345)
Supplement: Supplementary file 1 [file DataSheet_1.pdf]

SUPPLEMENTARY FIGURES:

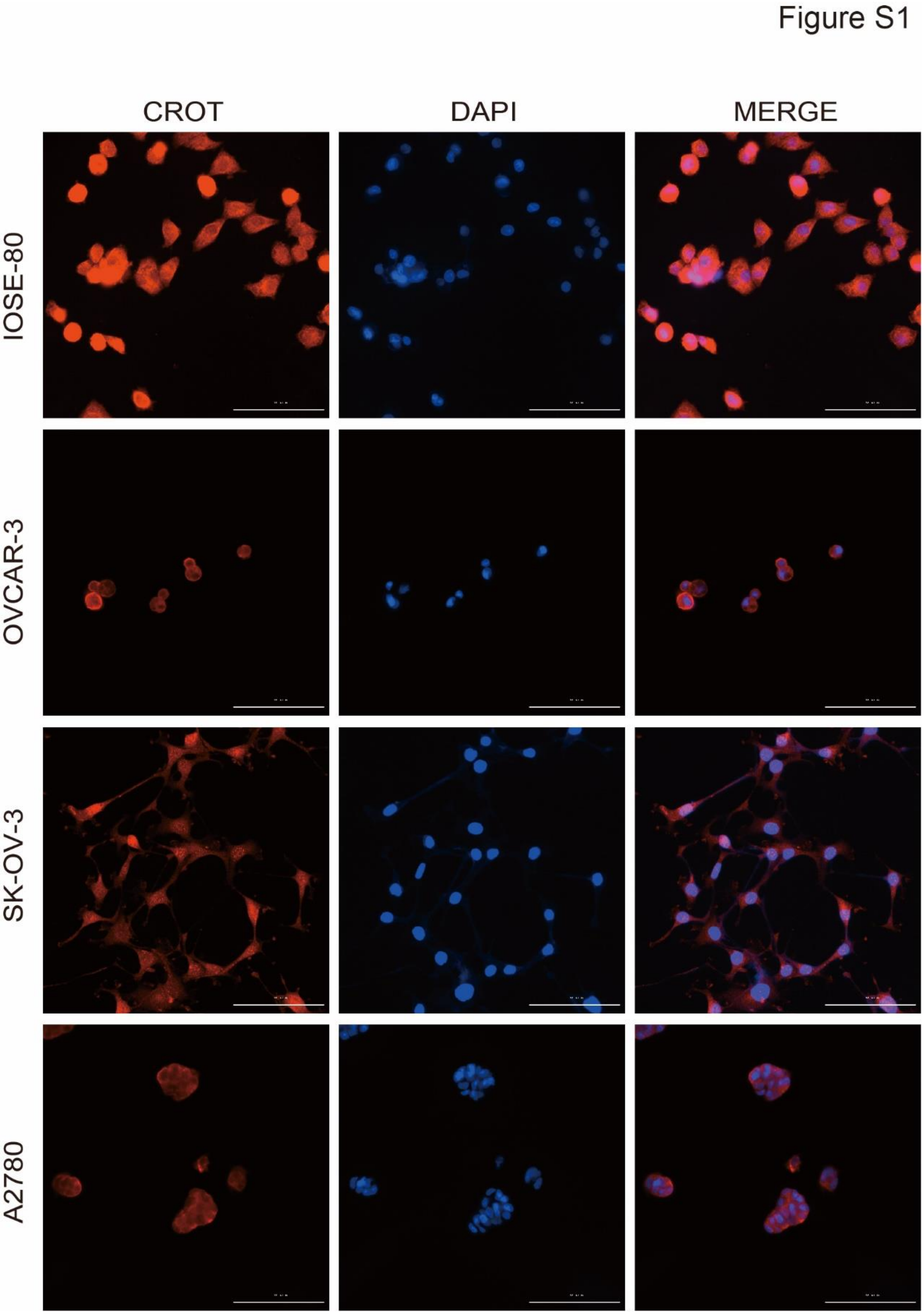

FIGURE S1 | Representative images of CROT in immortalized ovarian surface epithelium cell lines-80 (IOSE-80) and epithelial ovarian cancer cell lines (OVCAR-3, SK-OV-3, and A2780) by immunofluorescence. Original magnification, ×400; Scale bar, 100 μm.

Figure S2

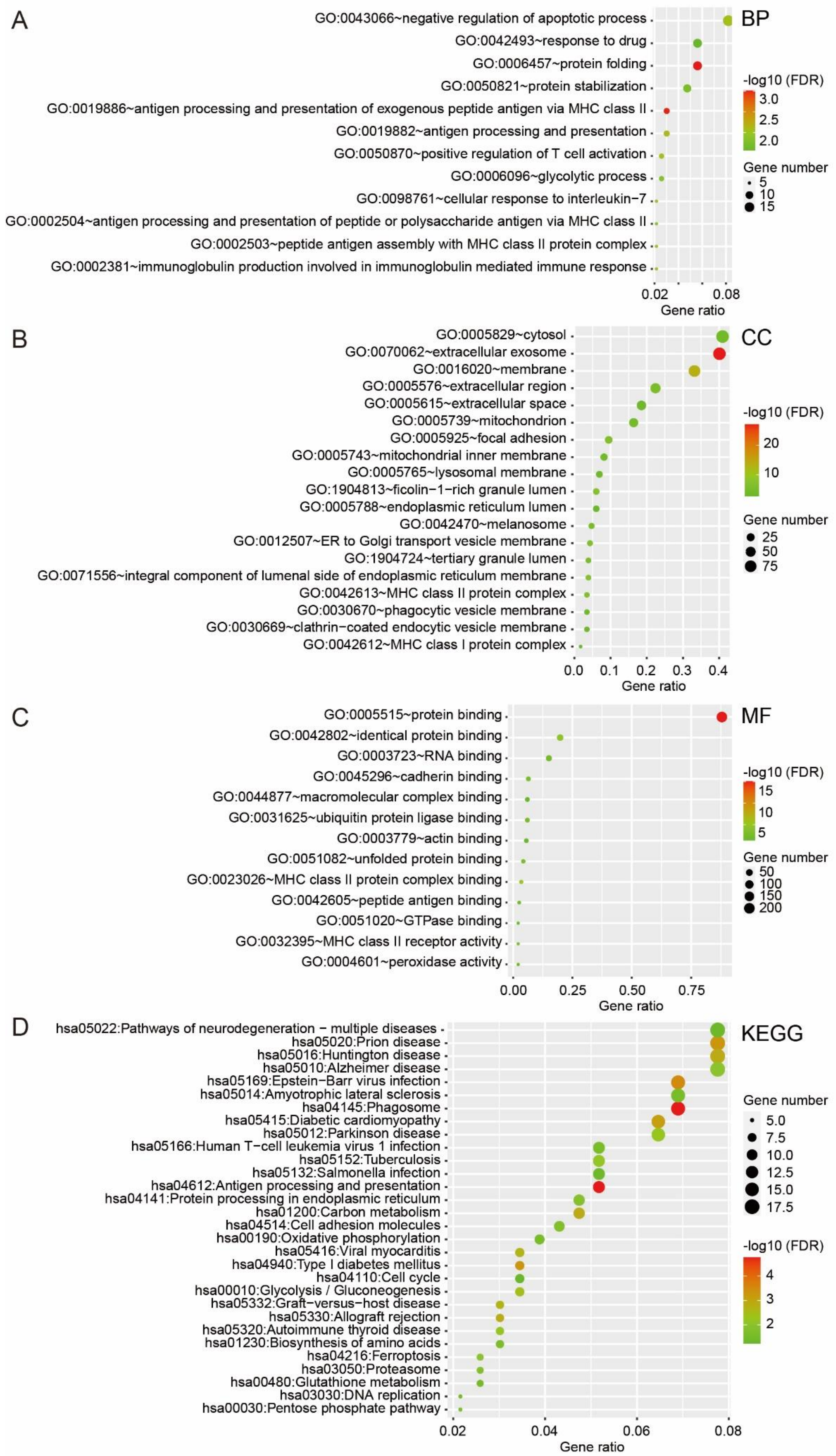
A
BP
GO:0043066~negative regulation of apoptotic process
GO:0042493~response to drug
GO:0006457~protein folding
GO:0050821~protein stabilization
GO:0019886~antigen processing and presentation of exogenous peptide antigen via MHC class II
GO:0019882~antigen processing and presentation
GO:0050870~positive regulation of T cell activation
GO:0006096~glycolytic process
GO:0098761~cellular response to interleukin−7
GO:0002504~antigen processing and presentation of peptide or polysaccharide antigen via MHC class II
GO:0002503~peptide antigen assembly with MHC class II protein complex
GO:0002381~immunoglobulin production involved in immunoglobulin mediated immune response
0.02
0.08
Gene ratio
-log10 (FDR)
3.0
2.5
2.0
Gene number
5
10
15
B
CC
GO:0005829~cytosol
GO:0070062~extracellular exosome
GO:0016020~membrane
GO:0005576~extracellular region
GO:0005615~extracellular space
GO:0005739~mitochondrion
GO:0005925~focal adhesion
GO:0005743~mitochondrial inner membrane
GO:0005765~lysosomal membrane
GO:1904813~ficolin−1−rich granule lumen
GO:0005788~endoplasmic reticulum lumen
GO:0042470~melanosome
GO:0012507~ER to Golgi transport vesicle membrane
GO:1904724~tertiary granule lumen
GO:0071556~integral component of lumenal side of endoplasmic reticulum membrane
GO:0042613~MHC class II protein complex
GO:0030670~phagocytic vesicle membrane
GO:0030669~clathrin−coated endocytic vesicle membrane
GO:0042612~MHC class I protein complex
0.0
0.1
0.2
0.3
0.4
Gene ratio
-log10 (FDR)
20
10
Gene number
25
50
75
C
MF
GO:0005515~protein binding
GO:0042802~identical protein binding
GO:0003723~RNA binding
GO:0045296~cadherin binding
GO:0044877~macromolecular complex binding
GO:0031625~ubiquitin protein ligase binding
GO:0003779~actin binding
GO:0051082~unfolded protein binding
GO:0023026~MHC class II protein complex binding
GO:0042605~peptide antigen binding
GO:0051020~GTPase binding
GO:0032395~MHC class II receptor activity
GO:0004601~peroxidase activity
0.00
0.25
0.50
0.75
Gene ratio
-log10 (FDR)
15
10
5
Gene number
50
100
150
200
D
KEGG
hsa05022:Pathways of neurodegeneration − multiple diseases
hsa05020:Prion disease
hsa05016:Huntington disease
hsa05010:Alzheimer disease
hsa05169:Epstein−Barr virus infection
hsa05014:Amyotrophic lateral sclerosis
hsa04145:Phagosome
hsa05415:Diabetic cardiomyopathy
hsa05012:Parkinson disease
hsa05166:Human T−cell leukemia virus 1 infection
hsa05152:Tuberculosis
hsa05132:Salmonella infection
hsa04612:Antigen processing and presentation
hsa04141:Protein processing in endoplasmic reticulum
hsa01200:Carbon metabolism
hsa04514:Cell adhesion molecules
hsa00190:Oxidative phosphorylation
hsa05416:Viral myocarditis
hsa04940:Type I diabetes mellitus
hsa04110:Cell cycle
hsa00010:Glycolysis / Gluconeogenesis
hsa05332:Graft−versus−host disease
hsa05330:Allograft rejection
hsa05320:Autoimmune thyroid disease
hsa01230:Biosynthesis of amino acids
hsa04216:Ferroptosis
hsa03050:Proteasome
hsa00480:Glutathione metabolism
hsa03030:DNA replication
hsa00030:Pentose phosphate pathway
0.02
0.04
0.06
0.08
Gene ratio
Gene number
5.0
7.5
10.0
12.5
15.0
17.5
-log10 (FDR)
4
3
2

FIGURE S2 | GO term and KEGG enrichment analyses of CROT in OC. (**A**) GO term annotation of biological processes (BP). (**B**) GO term annotation of cellular components (CC) based on the data from GSE63885. (**C**) GO term annotation of molecular function (MF) analysis. (**D**). Kyoto Encyclopedia of Genes and Genomes (KEGG) analysis of co-expression genes with CROT. The data were acquired from the GSE63885 dataset.

Figure S3

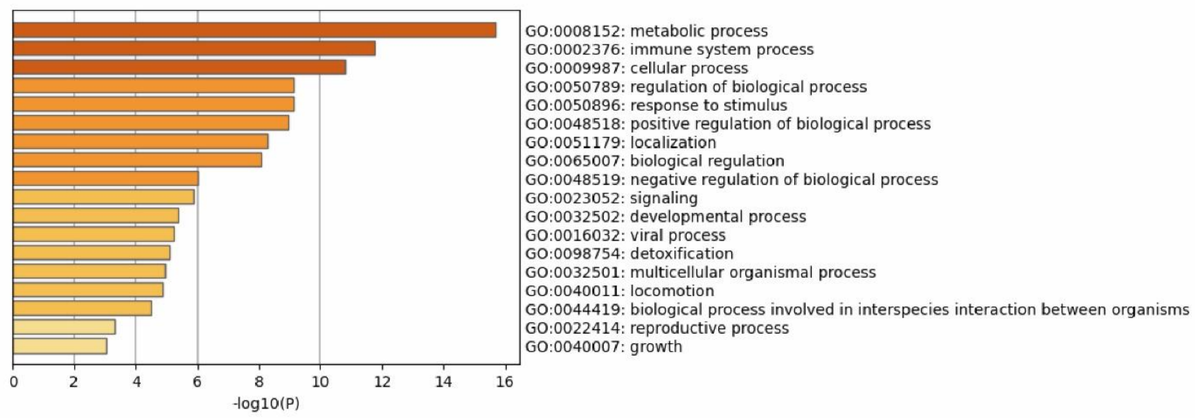

FIGURE S3 | Metascape analysis of co-expression genes with CROT. The data were acquired from the GSE26712 dataset (https://metascape.org/gp/index.html#/main/step1).

Figure S4

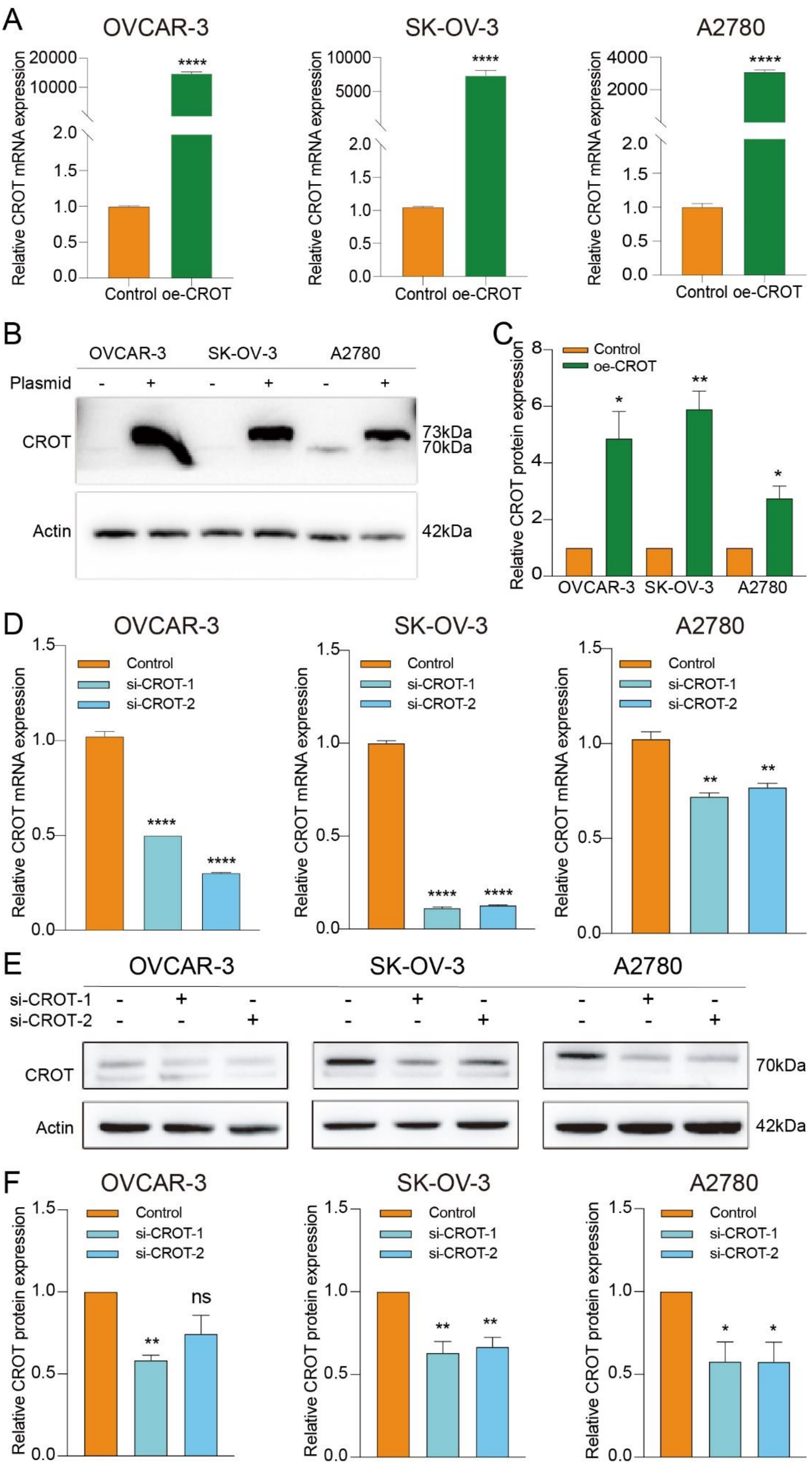

A
OVCAR-3
SK-OV-3
A2780
Relative CROT mRNA expression
Control
oe-CROT
****
B
Plasmid
CROT
Actin
73kDa
70kDa
42kDa
C
Control
oe-CROT
Relative CROT protein expression
D
si-CROT-1
si-CROT-2
E
F
ns

FIGURE S4 | Detection of CROT mRNA and protein expression by qRT-PCR and Western blot. (**A-C**) Expression of CROT mRNA and protein in OVACR-3, SK-OV-3, and A2780 cells after CROT-overexpressing plasmid (oe-CROT) transfection for 48 h. (**D-F**) Expression of CROT mRNA and protein detected in OVACR-3, SK-OV-3, and A2780 cells after CROT-siRNAs (si-CROT) transfection for 48 h. Assays were repeated at least three times. Data were presented as mean ± SEM. *, P <0.05; **, P < 0.01; ****, P < 0.0001; ns, not significant.

Figure S5

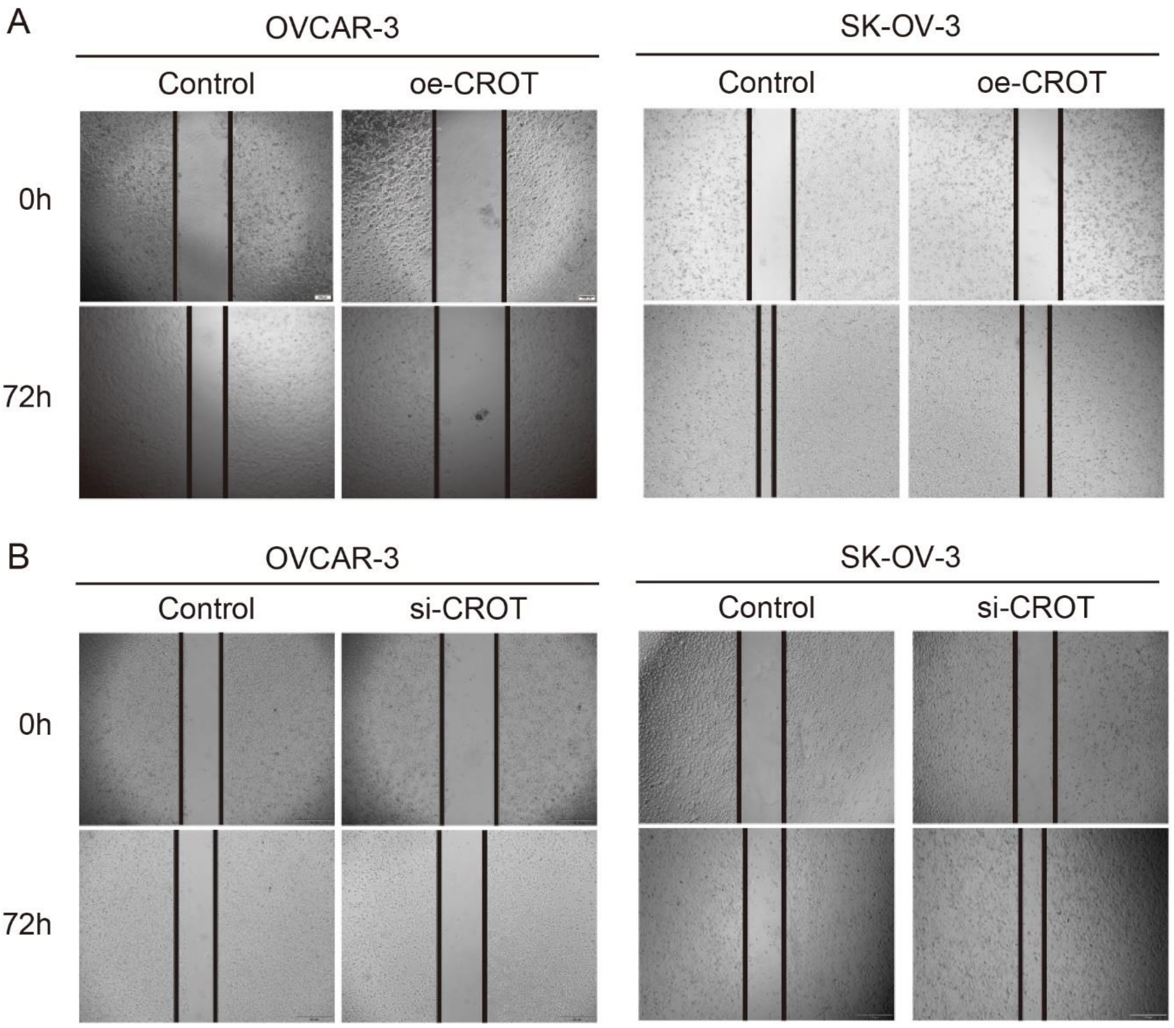

FIGURE S5 | Detection of cell migration by the wound healing assay. (**A**) Measurement of wound healing in OVCAR-3 and SK-OV-3 cells after CROT-overexpressing plasmid (oe-CROT) transfection for 0 and 72 h. (**B**) Measurement of wound healing in OVCAR-3 and SK-OV-3 cells after CROT-siRNAs (si-CROT) transfection for 0 and 72 h. Negative controls were compared. Scale bar, 100 μm.

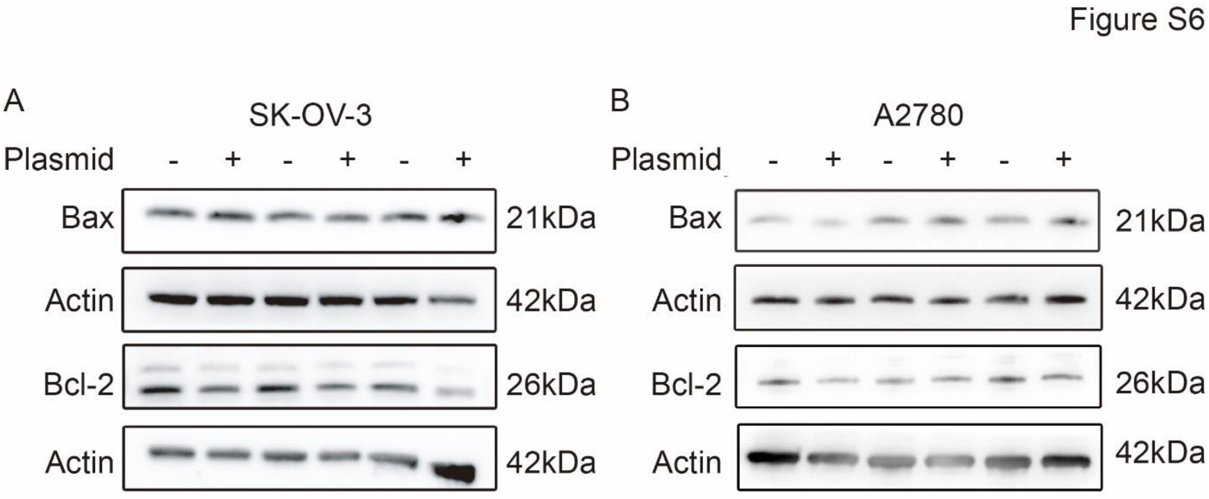

FIGURE S6 | Detection of Bax and Bcl-2 proteins by Western blot. (**A**-**B**) SK-OV-3 cells and A2780 cells were transfected with CROT-overexpressing plasmid (oe-CROT) for 48 h.

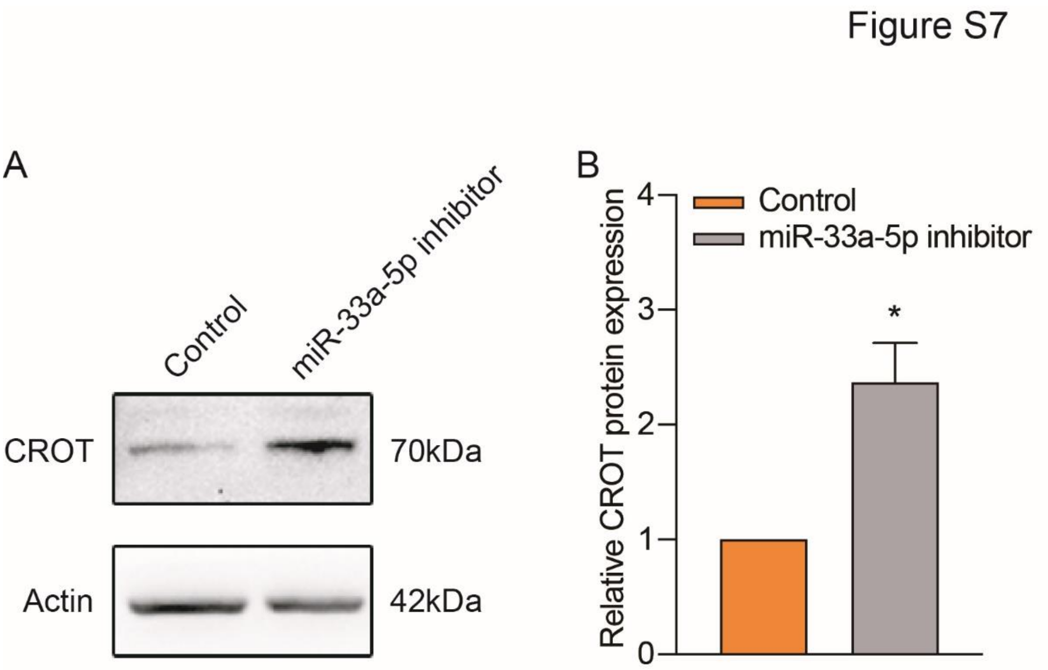

FIGURE S7 | Detection of CROT protein in SK-OV-3 cells. (**A**) Protein was detected by Western blot after the treatment of miR-33a-5p inhibitors for 48 h. (**B**) The histogram shows the densitometric analyses of protein bands in A (n=3). Data were presented as mean ± SEM. *, $P < 0.05$.

Figure S8

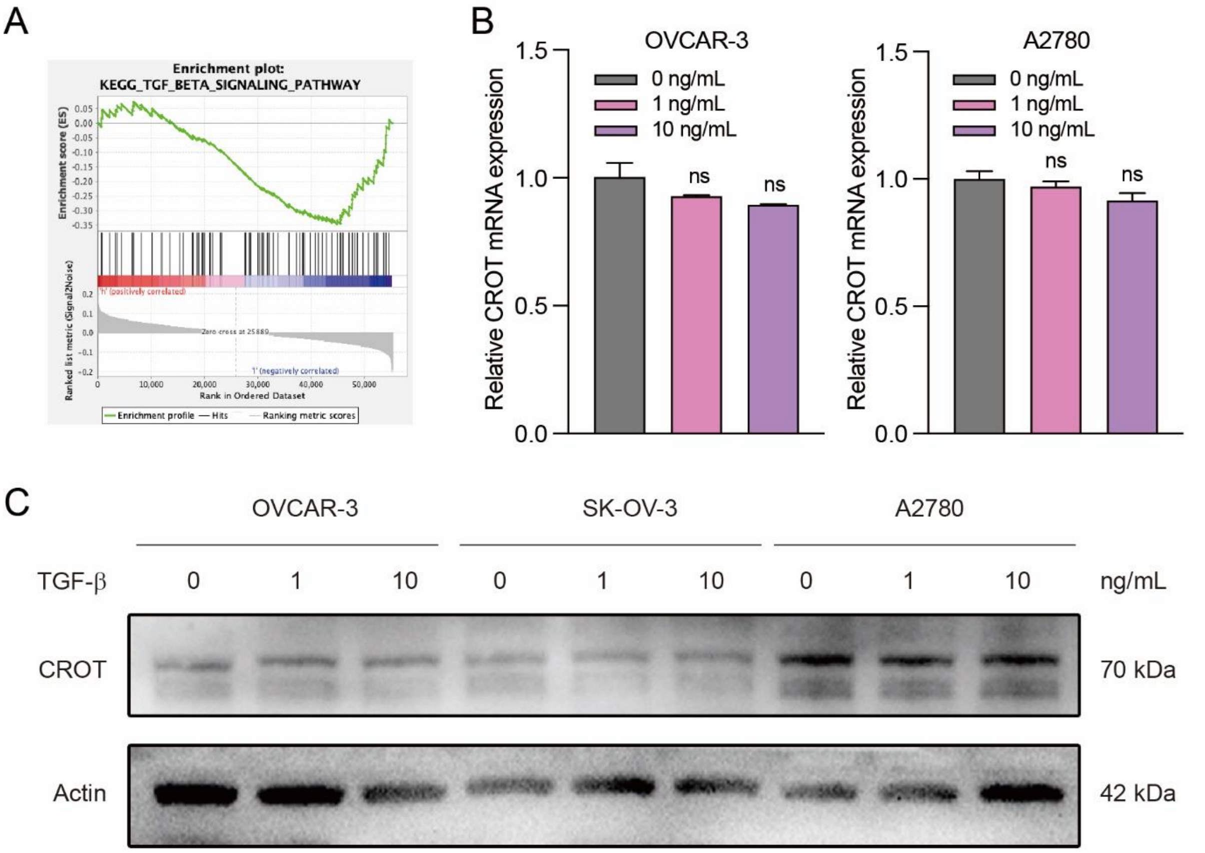

FIGURE S8 | Effect of TGF-β on CROT expression. (**A**) GSEA analysis of the association between the enrichment of the TGF-β signaling pathway and CROT expression based on the data from the TCGA-OV database. (**B-C**) Detection of CROT mRNA and protein expression in OVACR-3 and A2780 cells after the administration of TGF-β1 (0, 1, or 10 ng/ml) by qRT-PCR and Western blot. Data were presented as mean ± SEM. GSEA, gene set enrichment analysis; ns, not significant.
