# Supplementary material for: The miR-33a-5p/CROT axis mediates ovarian cancer cell behaviors and chemoresistance via the regulation of the TGF-β signal pathway
Source: Front Endocrinol (Lausanne). 2022 Sep 2;13:950345. doi: 10.3389/fendo.2022.950345 (PMC9478117; doi:10.3389/fendo.2022.950345)
Supplement: Supplementary file 2 [file Table_1.pdf]

SUPPLEMENTARY TABLES:

Table S1. Sequences of siRNA and miRNA

Items	Sequences	Position
siRNA		
CROT-siRNA-1	GCCUGUUCAUAAAGUUGGAAAtt	648-668
CROT-siRNA-2	CCAUCCUUAUUGGAGAUCCAAtt	1102-1123
NC-siRNA	UUCUCCGAACGUCGUCAGUtt	Scramble
miRNA		
miR-33a-5p mimics	GUGCAUUGUAGUUGCAUUGCA	
NC-mimics	UUGUACUACACAAAAGUACUG	Scramble
miR-33a-5p inhibitors	UGCAAUGCAACUACAAUGCAC	
NC-inhibitors	CAGUACUUUUGUGUAGUACAA	Scramble

GenBank accession number of CROT: NM_021151.4. NC, non-specific control.

Table S2. Sequences of RT-PCR primers

Genes	Sequences
miR-33a-5p-RT	GTCGTATCCAGTGCAGGGTCCGAGGTATTCGCACTGG ATACGACtgcaat
miR-33a-5p-F	GUGCAUUGUAGUUGCAUUGCA
miR-33a-5p-R	GGGCCTATTTCCCATGATTC
U6-F	GCTTCGGCAGCACATATACT
U6-R	GGGCCTATTTCCCATGATTC
CROT-F	GTGGTGGCTGAATGTTGCCTA
CROT-R	TTGGAGGCCAGTAGTGTTCAA
Actin-F	TCATCACCATTGGCAATGAG
Actin-R	CACTGTGTTGGCGTACAGGT

F, forward primer; R, reverse primer.

Table S3. Information of the GEO datasets involved in this analysis

GEO series	Contributors, Year	EOC (n)	Normal (n)	GEO Platform #
GSE26712	Birrer MJ, 2011	185	10	GPL96
GSE18521	Mok SC etc, 2009	53	10	GPL570
GSE40595	Birrer MJ, 2014	32	6	GPL570
GSE38666	Lili LN etc, 2012	18	12	GPL570
GSE14407	Bowen NJ etc, 2009	12	12	GPL570
GSE52460	Hill CG etc, 2013	10	10	GPL570

Samples used for the analysis of CROT expression were extracted from different GEO datasets. EOC, epithelial ovarian cancer; GEO, Gene Expression Omnibus; GPL, GEO Platform; n, number of samples; Normal, normal epithelial tissue of the ovary; n, number of samples.
